# Crystal structure of 1-methyl-2-[(*E*)-2-(4-methyl­phen­yl)ethen­yl]-4-nitro-1*H*-imidazole

**DOI:** 10.1107/S1600536814017243

**Published:** 2014-08-01

**Authors:** Hayette Alliouche, Abdelmalek Bouraiou, Sofiane Bouacida, Hocine Merazig, Ali Belfaitah

**Affiliations:** a Laboratoire des Produits Naturels d’Origine Végétale et de Synthèse Organique, PHYSYNOR, Université Constantine 1, 25000 Constantine, Algeria; b Unité de Recherche de Chimie de l’Environnement et Moléculaire Structurale (CHEMS), Université Constantine 1, 25000 , Algeria; c Département Sciences de la Matière, Faculté des Sciences Exactes et Sciences de la Nature et de la Vie, Université Oum El Bouaghi, Algeria

**Keywords:** crystal structure, imidazoles, nitro­imidazoles, pharmacophore, hydrogen bonding, π–π stacking inter­actions

## Abstract

In the title mol­ecule, C_13_H_13_N_3_O_2_, the planes of the benzene and imidazole rings form a dihedral angle of 7.72 (5)°. In the crystal, mol­ecules are linked by weak C—H⋯N and C—H⋯O hydrogen bonds, forming layers parallel to (100). A weak C—H⋯π inter­action connects these layers into a three-dimensional network. A π–π stacking inter­action, with a centroid–centroid distance of 3.5373 (9) Å, is also observed.

## Related literature   

For the synthesis and applications of imidazole derivatives, see: Mamedov *et al.* (2011 ▶); De Luca (2006 ▶); Teimouri & Chermahini (2011 ▶); Achar *et al.* (2010 ▶); Özkay *et al.* (2010 ▶); Shingalapur *et al.* (2009 ▶); Bhatia & Shanbhag (1984 ▶); Hoffer & Grunberg (1974 ▶). For the biological activity of nitro­imidazole derivatives, see: Trivedi *et al.* (2011 ▶); Leitsch *et al.* (2011 ▶); Luo *et al.* (2010 ▶); Saadeh *et al.* (2009 ▶); Thompson *et al.* (2009 ▶); Carvalho *et al.* (2006 ▶); Alliouche *et al.* (2014 ▶); Hunkeler *et al.* (1981 ▶); Tanigawara *et al.* (1999 ▶).
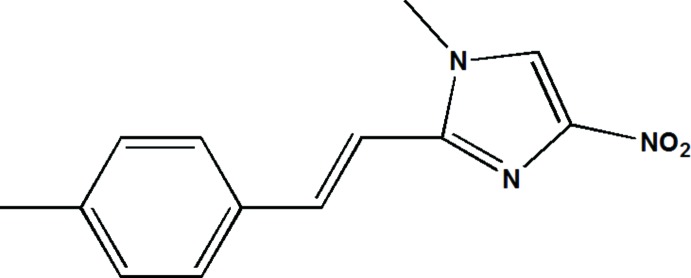


## Experimental   

### Crystal data  

C_13_H_13_N_3_O_2_
*M* *_r_* = 243.26
Monoclinic, 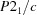

*a* = 7.1774 (8) Å
*b* = 15.7931 (16) Å
*c* = 10.7901 (11) Å
β = 101.798 (6)°
*V* = 1197.3 (2) Å^3^
*Z* = 4
Mo *K*α radiation
μ = 0.09 mm^−1^
*T* = 150 K
0.16 × 0.06 × 0.05 mm

### Data collection  

Bruker APEXII diffractometer
Absorption correction: multi-scan (*SADABS*; Sheldrick, 2002 ▶) *T*_min_ = 0.891, *T*_max_ = 1.000
9856 measured reflections
2113 independent reflections
1958 reflections with *I* > 2σ(*I*)
*R*_int_ = 0.025

### Refinement  

*R*[*F* ^2^ > 2σ(*F* ^2^)] = 0.035
*wR*(*F* ^2^) = 0.094
*S* = 1.05
2113 reflections
165 parameters
H-atom parameters constrained
Δρ_max_ = 0.36 e Å^−3^
Δρ_min_ = −0.31 e Å^−3^

### 

Data collection: *APEX2* (Bruker, 2006 ▶); cell refinement: *SAINT* (Bruker, 2006 ▶); data reduction: *SAINT*; program(s) used to solve structure: *SIR2002* (Burla *et al.*, 2005 ▶); program(s) used to refine structure: *SHELXL97* (Sheldrick, 2008 ▶); molecular graphics: *ORTEP-3 for Windows* (Farrugia, 2012 ▶) and *DIAMOND* (Brandenburg & Berndt, 2001 ▶); software used to prepare material for publication: *WinGX* (Farrugia, 2012 ▶).

## Supplementary Material

Crystal structure: contains datablock(s) I. DOI: 10.1107/S1600536814017243/lh5721sup1.cif

Structure factors: contains datablock(s) I. DOI: 10.1107/S1600536814017243/lh5721Isup2.hkl

Supporting information file. DOI: 10.1107/S1600536814017243/lh5721Isup3.cml

**Fig. 1.**
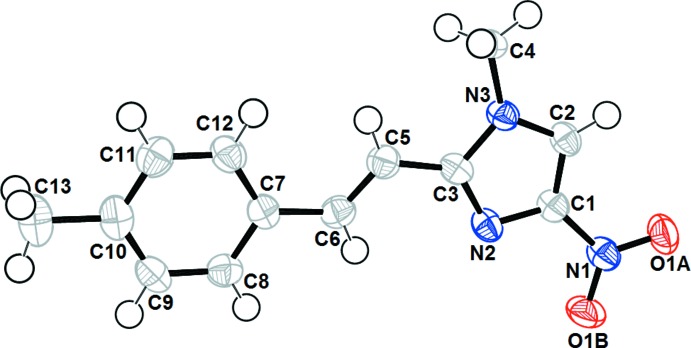
The molecular structure structure of the title compound with displacement ellipsoids drawn at the 50% probability level.

**Fig. 2.**
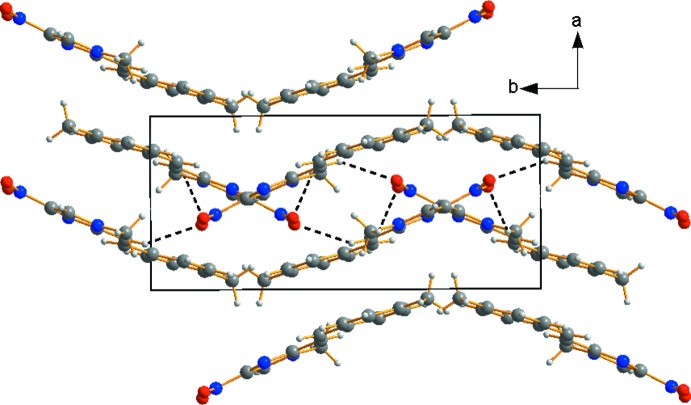
The crystal packing of (I) viewed along the c axis showing weak C—H···O hydrogen bonds as dashed lines.

**Fig. 3.**
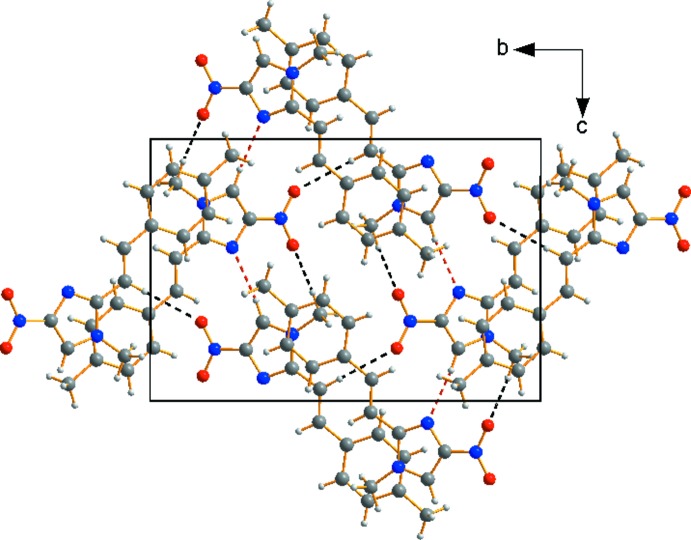
The crystal packing of (I) viewed along the a axis showing weak C—H···O and C—H···N hydrogen bonds as dashed lines.

. DOI: 10.1107/S1600536814017243/lh5721fig1.tif
The mol­ecular structure structure of the title compound with displacement ellipsoids drawn at the 50% probability level.

c . DOI: 10.1107/S1600536814017243/lh5721fig2.tif
The crystal packing of (I) viewed along the *c* axis showing weak C—H⋯O hydrogen bonds as dashed lines.

a . DOI: 10.1107/S1600536814017243/lh5721fig3.tif
The crystal packing of (I) viewed along the *a* axis showing weak C—H⋯O and C—H⋯N hydrogen bonds as dashed lines.

CCDC reference: 1016150

Additional supporting information:  crystallographic information; 3D view; checkCIF report

## Figures and Tables

**Table 1:** Hydrogen-bond geometry (Å, °) *Cg* is the centroid of the C7–C12 ring.

*D*—H⋯*A*	*D*—H	H⋯*A*	*D*⋯*A*	*D*—H⋯*A*
C2—H2⋯N2^i^	0.93	2.49	3.3702 (17)	159
C4—H4*C*⋯O1*B* ^i^	0.96	2.54	3.2676 (17)	133
C13—H13*B*⋯O1*A* ^ii^	0.96	2.59	3.5347 (19)	168
C4—H4*B*⋯*Cg* ^iii^	0.96	2.61	3.4336 (16)	144

Symmetry codes: (i) 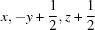; (ii) 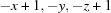; (iii) 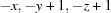.

## supplementary crystallographic information

### S1. Comment

The imidazole nucleus is an important pharmacophore found in a large number of natural products and synthetic compounds with a wide range of applications which make imidazole derivatives a subject of extensive investigations (Mamedov, *et al.*, 2011; De Luca, 2006; Tanigawara, *et al.*, 1999; Hunkeler, *et al.*, 1981). For example, many synthetic imidazole derivatives are present in a number of bioactive compounds such as fungicides, herbicides, bactericides, anti-inflammators, analgesics and anticancers (Teimouri, *et al.*, 2011; Achar, *et al.*, 2010; Özkay, *et al.*, 2010; Shingalapur, *et al.*, 2009). Nitroimidazoles are a particular class of imidazoles principally composed of bioactive substances where their spectrum of action is closely associated with the position of the nitro group on the imidazole ring. Due to their significant biological activity, 5-nitroimidazoles are widely used in medicine as bactericide and parasiticide agents, some of them possess an original activity spectrum especially towards protozoa and strict anaerobic bacteria (Trivedi, *et al.*, 2011; Bhatia, *et al.*, 1984; Hoffer *et al.*, 1974), and others exhibit cytotoxic and radiosensitization activities *in vitro* and *in vivo* (Leitsch, *et al.*, 2011; Luo, *et al.*, 2010). However, only few biological properties of 4-nitroimidazoles have been reported in the literature (Saadeh, *et al.*, 2009; Thompson, *et al.*, 2009; Carvalho, *et al.*, 2006). The transposition of a nitro group in 5-nitroimidazoles is a known reaction and constitutes an efficient synthetic procedure of 4-nitroisomers. However, only few examples of this reaction are described using methyl iodide as catalyst (Alliouche, *et al.* 2014). We report in this paper, the synthesis and structure determination of (*E*)-1-methyl-2-[(4-methylphenyl)-1-ethenyl]-5-nitroimidazole (I). The later was easily prepared from its 5-nitro isomer *via* an intramolecular transposition of the nitro group. The reaction was carried out in nitrobenzene at 433K using catalytic amount of methyl iodide.

The molecular structure of (I) is shown in Fig. 1. The benzene and imidazole ring form a dihedral angle of 7.72 (5)°. The crystal packing can be described as double zig-zag layers parallel to (100) (Fig. 2) which are stabilized by weak C—H···N and C—H···O hydrogen bonds. A weak C—H···π interaction links the layers forming a three-dimensional network (Fig. 2 and Fig. 3). The crystal structure features one π–π stacking interaction: *Cg*1—*Cg*2 (-x, -y, 1-z) = 3.5373 (9) Å Where, *Cg*1 is the centroid of the imidazole ring (C1/C2/N3/C3/N2) and *Cg*2 is is the centroid of the phenyl ring (C7/C8/C9/C10/C11/C12).

### S2. Experimental

The title compound (I) was obtained as yellow solid in 94% yield by heating the corresponding 5-nitroisomer at 433K in nitrobenzene in presence of methyl iodide as catalyst, during 24 h. Suitable crystals were obtained by slow evaporation of a solution of the title compound in water/methanol solution at room temperature.

### S3. Refinement

All non-H atoms were refined with anisotropic atomic displacement parameters. All H atoms were located in difference Fourier maps but were introduced in calculated positions and treated as riding on their parent atom (with C—H = 0.93 and 0.96 Å and *U*_iso_(H) = 1.5 or 1.2U_eq_(C).

### Crystal data

**Table:**

C_13_H_13_N_3_O_2_	*F*(000) = 512
*M**_r_* = 243.26	*D*_x_ = 1.35 Mg m^−^^3^
Monoclinic, *P*2_1_/*c*	Mo *K*α radiation, λ = 0.71073 Å
Hall symbol: -P 2ybc	Cell parameters from 6402 reflections
*a* = 7.1774 (8) Å	θ = 2.3–25.1°
*b* = 15.7931 (16) Å	µ = 0.09 mm^−^^1^
*c* = 10.7901 (11) Å	*T* = 150 K
β = 101.798 (6)°	Needle, colorless
*V* = 1197.3 (2) Å^3^	0.16 × 0.06 × 0.05 mm
*Z* = 4	

### Data collection

**Table:**

Bruker APEXII diffractometer	1958 reflections with *I* > 2σ(*I*)
Graphite monochromator	*R*_int_ = 0.025
CCD rotation images, thin slices scans	θ_max_ = 25.1°, θ_min_ = 2.6°
Absorption correction: multi-scan (*SADABS*; Sheldrick, 2002)	*h* = −8→8
*T*_min_ = 0.891, *T*_max_ = 1.000	*k* = −18→18
9856 measured reflections	*l* = −12→12
2113 independent reflections	

### Refinement

**Table:**

Refinement on *F*^2^	Primary atom site location: structure-invariant direct methods
Least-squares matrix: full	Secondary atom site location: difference Fourier map
*R*[*F*^2^ > 2σ(*F*^2^)] = 0.035	Hydrogen site location: inferred from neighbouring sites
*wR*(*F*^2^) = 0.094	H-atom parameters constrained
*S* = 1.05	*w* = 1/[σ^2^(*F*_o_^2^) + (0.0455*P*)^2^ + 0.712*P*] where *P* = (*F*_o_^2^ + 2*F*_c_^2^)/3
2113 reflections	(Δ/σ)_max_ = 0.005
165 parameters	Δρ_max_ = 0.36 e Å^−^^3^
0 restraints	Δρ_min_ = −0.31 e Å^−^^3^

### Special details

**Table:**

Geometry. All e.s.d.'s (except the e.s.d. in the dihedral angle between two l.s. planes) are estimated using the full covariance matrix. The cell e.s.d.'s are taken into account individually in the estimation of e.s.d.'s in distances, angles and torsion angles; correlations between e.s.d.'s in cell parameters are only used when they are defined by crystal symmetry. An approximate (isotropic) treatment of cell e.s.d.'s is used for estimating e.s.d.'s involving l.s. planes.
Refinement. Refinement of *F*^2^ against ALL reflections. The weighted *R*-factor *wR* and goodness of fit *S* are based on *F*^2^, conventional *R*-factors *R* are based on *F*, with *F* set to zero for negative *F*^2^. The threshold expression of *F*^2^ > σ(*F*^2^) is used only for calculating *R*-factors(gt) *etc*. and is not relevant to the choice of reflections for refinement. *R*-factors based on *F*^2^ are statistically about twice as large as those based on *F*, and *R*- factors based on ALL data will be even larger.

### Fractional atomic coordinates and isotropic or equivalent isotropic displacement parameters (Å^2^)

**Table:**

	*x*	*y*	*z*	*U*_iso_*/*U*_eq_	
N2	0.41984 (15)	0.20989 (7)	0.58571 (10)	0.0142 (3)	
O1B	0.58852 (16)	0.36479 (6)	0.59448 (9)	0.0234 (3)	
O1A	0.62422 (14)	0.36871 (6)	0.79969 (9)	0.0214 (3)	
N3	0.37372 (15)	0.13617 (7)	0.75347 (10)	0.0136 (3)	
N1	0.56745 (16)	0.33418 (7)	0.69608 (10)	0.0154 (3)	
C12	0.16578 (19)	−0.08459 (9)	0.37141 (12)	0.0173 (3)	
H12	0.1714	−0.0947	0.457	0.021*	
C1	0.47447 (18)	0.25439 (8)	0.69479 (12)	0.0138 (3)	
C7	0.20697 (18)	−0.00361 (8)	0.33138 (12)	0.0148 (3)	
C3	0.35902 (18)	0.13706 (8)	0.62364 (12)	0.0136 (3)	
C9	0.15285 (19)	−0.05791 (9)	0.11606 (13)	0.0177 (3)	
H9	0.1512	−0.0485	0.0308	0.021*	
C5	0.29083 (19)	0.06490 (8)	0.54450 (12)	0.0157 (3)	
H5	0.265	0.0148	0.5831	0.019*	
C2	0.44778 (18)	0.21119 (8)	0.79988 (12)	0.0141 (3)	
H2	0.4744	0.2293	0.8837	0.017*	
C11	0.11685 (19)	−0.14954 (9)	0.28511 (13)	0.0187 (3)	
H11	0.0879	−0.2025	0.3138	0.022*	
C6	0.26340 (18)	0.06724 (8)	0.41831 (12)	0.0157 (3)	
H6	0.2821	0.1192	0.3821	0.019*	
C10	0.10965 (19)	−0.13770 (9)	0.15583 (13)	0.0182 (3)	
C4	0.3300 (2)	0.06497 (8)	0.82950 (12)	0.0169 (3)	
H4A	0.4249	0.0217	0.8324	0.025*	
H4B	0.2073	0.0424	0.7921	0.025*	
H4C	0.3293	0.0841	0.9139	0.025*	
C13	0.0545 (2)	−0.20885 (10)	0.06267 (14)	0.0255 (3)	
H13A	−0.0782	−0.2215	0.055	0.038*	
H13B	0.1287	−0.2582	0.092	0.038*	
H13C	0.0779	−0.1923	−0.0184	0.038*	
C8	0.19843 (19)	0.00802 (9)	0.20157 (12)	0.0168 (3)	
H8	0.224	0.0613	0.1722	0.02*	

### Atomic displacement parameters (Å^2^)

**Table:**

	*U*^11^	*U*^22^	*U*^33^	*U*^12^	*U*^13^	*U*^23^
N2	0.0171 (6)	0.0135 (6)	0.0119 (5)	0.0024 (4)	0.0024 (4)	−0.0006 (4)
O1B	0.0390 (6)	0.0180 (5)	0.0150 (5)	−0.0046 (4)	0.0093 (4)	0.0023 (4)
O1A	0.0312 (6)	0.0174 (5)	0.0144 (5)	−0.0033 (4)	0.0019 (4)	−0.0040 (4)
N3	0.0162 (6)	0.0129 (6)	0.0118 (5)	0.0014 (4)	0.0033 (4)	0.0011 (4)
N1	0.0199 (6)	0.0129 (6)	0.0136 (6)	0.0026 (4)	0.0037 (4)	−0.0001 (4)
C12	0.0190 (7)	0.0184 (7)	0.0135 (6)	0.0014 (5)	0.0016 (5)	0.0007 (5)
C1	0.0164 (6)	0.0116 (6)	0.0135 (6)	0.0027 (5)	0.0034 (5)	−0.0002 (5)
C7	0.0122 (6)	0.0175 (7)	0.0147 (6)	0.0012 (5)	0.0024 (5)	−0.0013 (5)
C3	0.0137 (6)	0.0148 (7)	0.0123 (6)	0.0031 (5)	0.0028 (5)	0.0012 (5)
C9	0.0153 (7)	0.0249 (7)	0.0130 (6)	0.0016 (5)	0.0030 (5)	−0.0021 (5)
C5	0.0172 (7)	0.0139 (6)	0.0163 (7)	0.0003 (5)	0.0040 (5)	−0.0001 (5)
C2	0.0164 (6)	0.0134 (6)	0.0126 (6)	0.0028 (5)	0.0028 (5)	−0.0015 (5)
C11	0.0182 (7)	0.0150 (7)	0.0216 (7)	0.0007 (5)	0.0014 (5)	0.0013 (5)
C6	0.0158 (7)	0.0142 (7)	0.0176 (7)	0.0002 (5)	0.0043 (5)	0.0010 (5)
C10	0.0126 (6)	0.0207 (7)	0.0202 (7)	0.0029 (5)	0.0008 (5)	−0.0055 (6)
C4	0.0211 (7)	0.0165 (7)	0.0136 (6)	−0.0008 (5)	0.0049 (5)	0.0029 (5)
C13	0.0260 (8)	0.0249 (8)	0.0238 (8)	−0.0004 (6)	0.0009 (6)	−0.0088 (6)
C8	0.0157 (7)	0.0181 (7)	0.0170 (7)	−0.0003 (5)	0.0043 (5)	0.0011 (5)

### Geometric parameters (Å, º)

**Table:**

N2—C3	1.3244 (17)	C9—C10	1.387 (2)
N2—C1	1.3581 (17)	C9—H9	0.93
O1B—N1	1.2354 (15)	C5—C6	1.3358 (19)
O1A—N1	1.2357 (15)	C5—H5	0.93
N3—C2	1.3522 (17)	C2—H2	0.93
N3—C3	1.3832 (16)	C11—C10	1.398 (2)
N3—C4	1.4631 (16)	C11—H11	0.93
N1—C1	1.4246 (17)	C6—H6	0.93
C12—C11	1.3816 (19)	C10—C13	1.5057 (19)
C12—C7	1.4006 (19)	C4—H4A	0.96
C12—H12	0.93	C4—H4B	0.96
C1—C2	1.3700 (18)	C4—H4C	0.96
C7—C8	1.4015 (19)	C13—H13A	0.96
C7—C6	1.4632 (18)	C13—H13B	0.96
C3—C5	1.4479 (18)	C13—H13C	0.96
C9—C8	1.3853 (19)	C8—H8	0.93
			
C3—N2—C1	103.69 (11)	N3—C2—H2	128
C2—N3—C3	108.00 (11)	C1—C2—H2	128
C2—N3—C4	125.37 (11)	C12—C11—C10	121.71 (13)
C3—N3—C4	126.51 (11)	C12—C11—H11	119.1
O1B—N1—O1A	123.54 (11)	C10—C11—H11	119.1
O1B—N1—C1	118.65 (11)	C5—C6—C7	126.59 (13)
O1A—N1—C1	117.80 (11)	C5—C6—H6	116.7
C11—C12—C7	120.71 (12)	C7—C6—H6	116.7
C11—C12—H12	119.6	C9—C10—C11	117.74 (12)
C7—C12—H12	119.6	C9—C10—C13	121.09 (13)
N2—C1—C2	113.28 (12)	C11—C10—C13	121.16 (13)
N2—C1—N1	121.19 (11)	N3—C4—H4A	109.5
C2—C1—N1	125.23 (12)	N3—C4—H4B	109.5
C12—C7—C8	117.33 (12)	H4A—C4—H4B	109.5
C12—C7—C6	123.28 (12)	N3—C4—H4C	109.5
C8—C7—C6	119.36 (12)	H4A—C4—H4C	109.5
N2—C3—N3	111.11 (11)	H4B—C4—H4C	109.5
N2—C3—C5	126.46 (12)	C10—C13—H13A	109.5
N3—C3—C5	122.40 (11)	C10—C13—H13B	109.5
C8—C9—C10	120.94 (12)	H13A—C13—H13B	109.5
C8—C9—H9	119.5	C10—C13—H13C	109.5
C10—C9—H9	119.5	H13A—C13—H13C	109.5
C6—C5—C3	122.75 (12)	H13B—C13—H13C	109.5
C6—C5—H5	118.6	C9—C8—C7	121.55 (13)
C3—C5—H5	118.6	C9—C8—H8	119.2
N3—C2—C1	103.93 (11)	C7—C8—H8	119.2
			
C3—N2—C1—C2	−0.46 (15)	C3—N3—C2—C1	0.06 (14)
C3—N2—C1—N1	173.57 (11)	C4—N3—C2—C1	176.18 (12)
O1B—N1—C1—N2	3.97 (18)	N2—C1—C2—N3	0.25 (15)
O1A—N1—C1—N2	−175.44 (11)	N1—C1—C2—N3	−173.49 (12)
O1B—N1—C1—C2	177.24 (12)	C7—C12—C11—C10	1.1 (2)
O1A—N1—C1—C2	−2.16 (19)	C3—C5—C6—C7	175.78 (12)
C11—C12—C7—C8	−0.70 (19)	C12—C7—C6—C5	2.8 (2)
C11—C12—C7—C6	−178.70 (12)	C8—C7—C6—C5	−175.12 (13)
C1—N2—C3—N3	0.48 (14)	C8—C9—C10—C11	−1.05 (19)
C1—N2—C3—C5	−177.57 (12)	C8—C9—C10—C13	178.22 (12)
C2—N3—C3—N2	−0.35 (14)	C12—C11—C10—C9	−0.2 (2)
C4—N3—C3—N2	−176.42 (11)	C12—C11—C10—C13	−179.50 (13)
C2—N3—C3—C5	177.79 (12)	C10—C9—C8—C7	1.5 (2)
C4—N3—C3—C5	1.73 (19)	C12—C7—C8—C9	−0.57 (19)
N2—C3—C5—C6	−7.0 (2)	C6—C7—C8—C9	177.51 (12)
N3—C3—C5—C6	175.11 (12)		

### Hydrogen-bond geometry (Å, º)

Cg is the centroid of the C7–C12 ring.

**Table:**

*D*—H···*A*	*D*—H	H···*A*	*D*···*A*	*D*—H···*A*
C2—H2···N2^i^	0.93	2.49	3.3702 (17)	159
C4—H4*C*···O1*B*^i^	0.96	2.54	3.2676 (17)	133
C13—H13*B*···O1*A*^ii^	0.96	2.59	3.5347 (19)	168
C4—H4*B*···*Cg*^iii^	0.96	2.61	3.4336 (16)	144

Symmetry codes: (i) *x*, −*y*+1/2, *z*+1/2; (ii) −*x*+1, −*y*, −*z*+1; (iii) −*x*, −*y*+1, −*z*+1.
