# Supplementary figures and images for: Crystal structure of 1-methyl-2-[(E)-2-(4-methyl­phen­yl)ethen­yl]-4-nitro-1H-imidazole
Source: Acta Crystallogr Sect E Struct Rep Online. 2014 Aug 1;70(Pt 9):o962–3. doi: 10.1107/S1600536814017243 (PMC4186084; doi:10.1107/S1600536814017243)

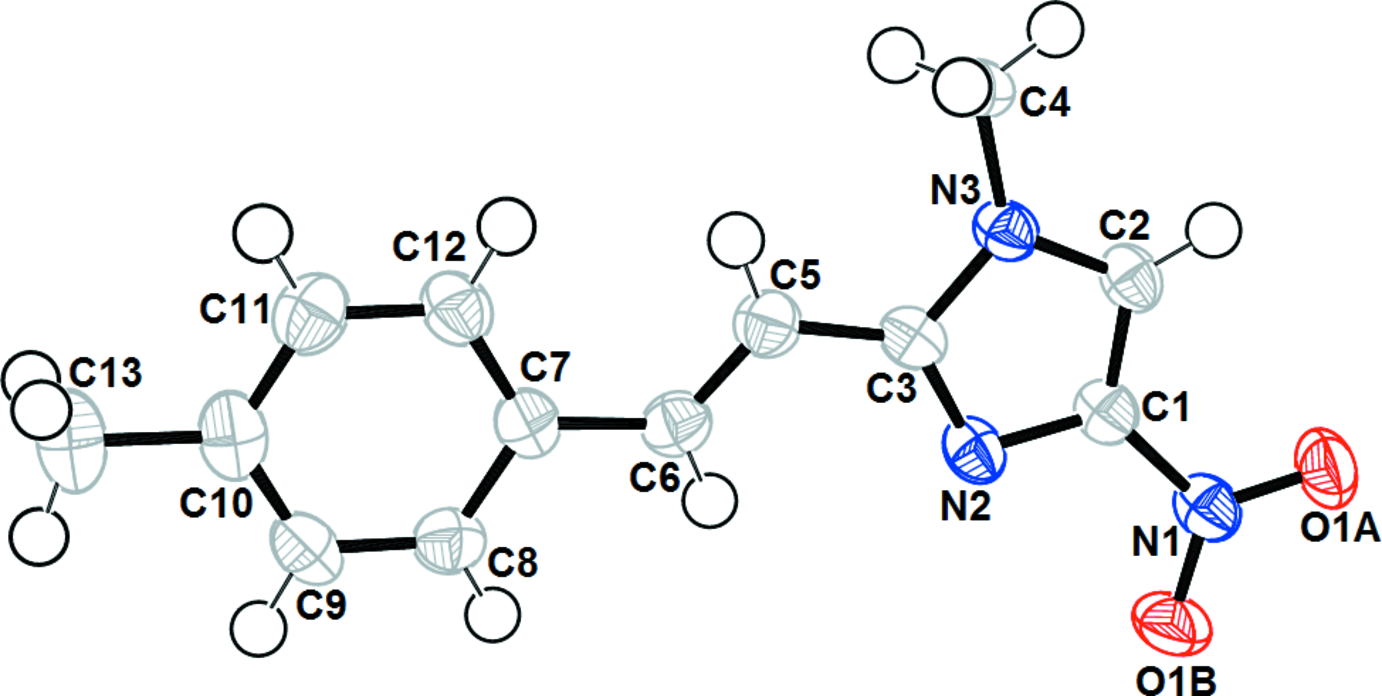

Supplement: Supplementary file 4 [file e-70-0o962-fig1.tif]

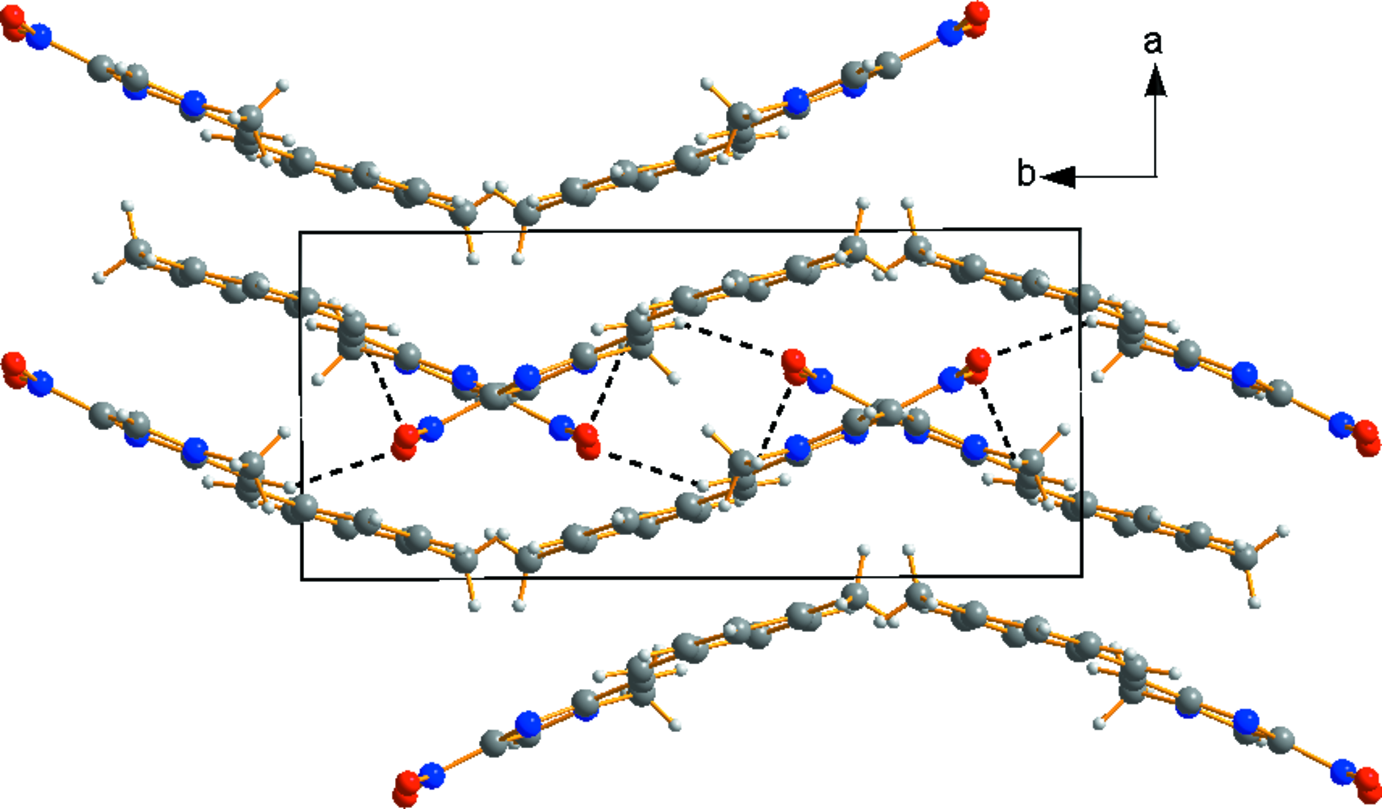

Supplement: Supplementary file 5 [file e-70-0o962-fig2.tif]

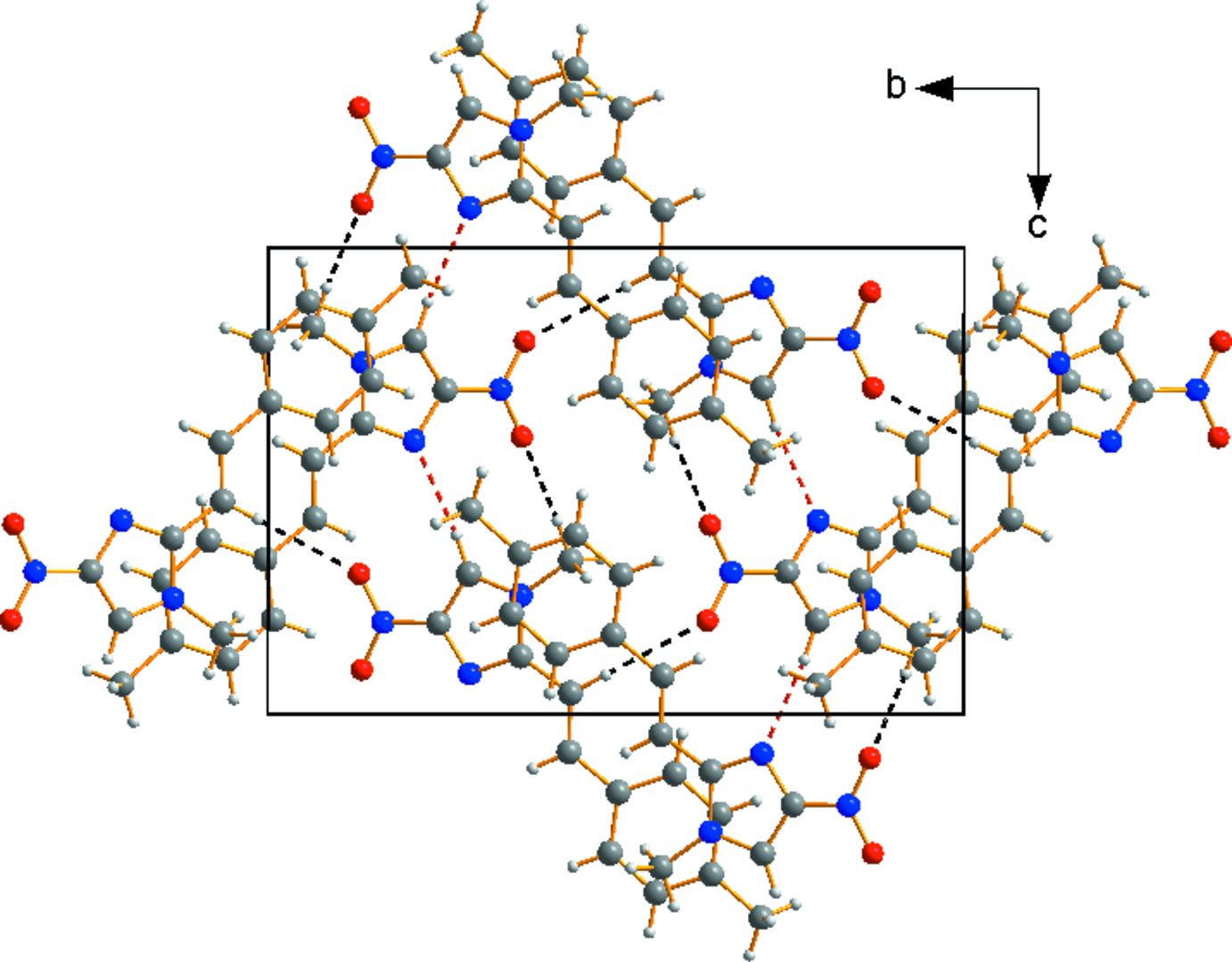

Supplement: Supplementary file 6 [file e-70-0o962-fig3.tif]
